# Management of fatigue in gynaecological cancer: A feasibility study of an app-based exercise and mindfulness intervention

**DOI:** 10.1016/j.gore.2025.101807

**Published:** 2025-07-19

**Authors:** Kairen McCloy, Ciara Hughes, Lynn Dunwoody, Joanne Marley, Ian Cleland, Federico Cruciani, Jackie Gracey

**Affiliations:** a Northern Health and Social Care Trust, UK; b Institute of Nursing and Health Research, Ulster University, Belfast, UK; c Psychology Research Institute, Ulster University, Coleraine, UK; d School of Computing, Ulster University, Belfast, UK

**Keywords:** Cancer, Exercise, Fatigue, Gynaecology, Mindfulness, Sleep, Quality of life

## Abstract

**Objectives:**

Cancer-related fatigue (CRF) is an issue for many people living with and beyond cancer. Evidence suggests that exercise and mindfulness may help in management of CRF, however, adherence to such interventions remains poor and there is little evidence for combining these interventions or for digital delivery. Few interventions have targeted women who have gynaecological cancer. This study developed and assessed the feasibility of digitally delivering mindfulness and exercise interventions to manage CRF in this population.

**Methods:**

An 8-week feasibility randomised control trial was delivered via a mobile app to two groups (mindfulness only; mindfulness and exercise). Feasibility was assessed through retention, adherence and attrition rates. Participant-reported outcome measures (PROMs) for fatigue, anxiety, depression, sleep and health-related quality of life were collected pre and post intervention and analysed using descriptive statistics (mean, median, inter-quartile ranges and line graphs). Online focus groups explored patient experiences, acceptability and satisfaction with the interventions.

**Results:**

Twenty-five participants (mindfulness only n = 13, mindfulness and exercise n = 12) had overall retention rate of 88 %, adherence was 72.72 % and attrition was 12 %. Both groups demonstrated improvements in fatigue (mindfulness-alone; 7.8, mindfulness and exercise 14.11), anxiety (2.9; 4.87), depression (2.06; 4.26), sleep, (2.8; 2.71), and HRQOL (6.4; 10.4), based on changes in mean scores. Qualitative findings identified three main themes: benefits of participation, barriers to participation and digital delivery of the intervention.

**Conclusion:**

Both groups experienced improvements in CRF, anxiety, depression, sleep and HRQoL. Retention and adherence rates were high with a good level of app engagement. Feasibility was demonstrated through retention, adherence and attrition rates and interventions were deemed acceptable. These findings suggest that a fully powered RCT is warranted. While online recruitment was challenging, the online delivery of interventions enabled broader participant inclusion and scalability.

## Highlights

• Digital delivery was satisfactory and acceptable but combination with health care professionals input was still preferable.
• Recruitment, retention and adherence were acceptable and therefore the study protocol is feasible.
• Despite scepticism, mindfulness alone had a positive effect on clinical outcomes of fatigue, sleep and quality of life.
• Reciprocal effect of combining interventions resulting in increase of activity participation which may lead to larger effects.
• Combination of mindfulness and exercise resulted in improvements in fatigue, sleep and QoL beyond MCID.

## 1 Background/Introduction

Globally in 2022 over 1. 4 million women were diagnosed with a gynaecological cancer, accounting for 15.25 % of all the cases of women with cancer. It is predicted that by 2050 this number will rise by 49.61 % with the number of new cases reaching 2 204 390 (Zhu et al., 2024). In the United Kingdom (UK) 21,493 new cases of gynaecological cancer are diagnosed every year (Gynaecological Cancer Research Charity., 2020). Treatment modalities such as surgery, chemotherapy or radiotherapy can be effective in managing and treating these cancers but may lead to unwanted long-term side effects. One of these is fatigue, which has a prevalence of 52 % across all types of cancer. For the gynaecological cancer population, this varied between 17–33 %, with many women experiencing cancer-related fatigue (CRF) years after treatment (Ma et al., 2020). CRF has been described not only as a physical sensation, but also has emotional and cognitive symptoms suggesting that it is multi-dimensional (de Raaf et al., 2013).

Risk factors for CRF, include depression and insomnia, suggesting that they are not only correlated, but may also impact on the levels and severity of CRF (Bower, 2014). Indeed, the term ‘cluster symptom’ has recently been adopted to describe, the most common symptoms of CRF, which are depression, insomnia, and fatigue (Lengacher et al., 2017). This suggests that managing CRF may require the incorporation of interventions that deal with the multiple symptoms in a more collective way. (Poort et al., 2020).

Currently research indicates that exercise can have positive effects on CRF (Donnelly et al., 2011, Sheehan et al., 2020). However, the effect of exercise on CRF and quality of life (QoL) remains unclear (Lin et al., 2016), some studies have reported positive outcomes and others demonstrating no change (Donnelly et al., 2011). This ambiguity may suggest that exercise alone may not be enough to ameliorate CRF and improve QoL and a multidimensional approach may be required.

More recently other interventions such as mindfulness have demonstrated a positive effect on both CRF and QoL (Johns et al., 2015, Lengacher et al., 2017). Mindfulness involves being intentionally aware of the present moment and doing this without judgment. Through practice, participants develop an awareness of current emotions and thoughts with compassion and kindness which in turn will lead to better control of cognitions, emotions, and behaviour (Park et al., 2020). However, effect sizes for mindfulness on psychological and physical outcomes such as depression, anxiety, and fatigue have been small, not reaching minimal clinical significance. Despite this, it seems that mindfulness had positive effects on psychological distress, which includes symptoms of anxiety, depression, sleep, stress and quality of life (McCloy et al., 2022). Some of these symptoms are also present within the ‘cluster’ previously identified as part of CRF, which suggests that mindfulness may be an appropriate intervention for reducing CRF.

Digital interventions for health have grown in number with over 50,000 medical or healthcare apps available on Google Play or the Apple Apps Store (Store, 2022). For people living with and beyond cancer, apps can provide education, help identify, and manage symptoms, encourage lifestyle change, and promote functional exercise (Cai et al., 2021). A recent review identified how digital and interactive health interventions had positive effects on the physical and psychological symptoms of women with breast cancer. Activities such as music therapy, walking, swimming, resistance training, dance, support and education were successfully delivered through a digital platform and women showed improvements in symptoms (Obrero-Gaitan et al., 2022). Mindfulness, when delivered digitally, has also been shown to be feasible and has resulted in positive outcomes (Lengacher et al., 2017). Therefore, evidence suggests that delivery of various interventions for cancer patients can be achieved remotely, increasing scale and inclusivity of a wider population.

The majority of studies investigating exercise or mindfulness for the management of CRF and psychological distress are in the breast cancer population making it difficult to generalise findings to other cancer populations (Stelten et al., 2020). Both exercise and mindfulness as standalone interventions have demonstrated positive effects on CRF, anxiety, depression, and sleep. However, whether mindfulness alone, or delivered in combination with exercise offers additional benefits for CRF management in women with gynaecological cancer remains unexplored. To address this the current study evaluated the feasibility, acceptability and clinical outcome of a digitally delivered intervention on CRF and its associated symptom cluster of anxiety, depression sleep and QoL in women with gynaecological cancer.

## 2 Methods

### 2.1 Design, setting and participants

An 8 week randomised controlled feasibility trial of mindfulness and home-based walking and strength training was compared to a mindfulness-only intervention. It was conducted remotely through a mobile application interface (app), which permitted recruitment across the UK and asynchronous delivery. The published study protocol provides a more detailed description of the study methods (McCloy et al., 2023). Recruitment took place via social media between June 2022 and February 2023 through Cancer Focus, Macmillan and Action Cancer. These organisations agreed to contact their service users through social media (Facebook, Twitter, Instagram), leaflet drops and other service providers the organisation provide such as counsellors, clinical nurse specialist, support workers. Ethical approval was obtained from the Research Ethics Committee at Ulster University in December 2021(REC21/0076). Eligibility criteria included women >18 years, with a diagnosis of gynaecological cancer (stage I-IV) who had completed treatment and were within 5 years of diagnosis. Participants had to have self-reported fatigue at level 4 or above, assessed on a 10-point single-item scale where 0=’no fatigue’ and 10=’greatest possible fatigue’ (Schwartz et al., 2002). Participants were a currently sedentary ((i.e. vigorous physical activity ≤20 mins/week or moderate physical activity ≤60 mins/week, for the past 6 months).

Participants were excluded if they were currently practising mindfulness or had a medical, psychiatric illness or fatigue-related co-morbidity that would inhibit safe participation in the study (i.e. fibromyalgia, arthritis, unstable cardiovascular disease, uncontrolled hypertension, or severe mental illness).

### 2.2 Procedures

Participants were screened for eligibility via telephone and written informed consent was obtained. Baseline outcomes were completed online via Qualtrics© prior to randomisation. Study randomisation was completed through Study Randomizer (2017) (Study Randomizer., 2017) a web-based randomisation service. Participants were randomly allocated to groups through 1:1 allocation using block randomisation with fixed blocks.

### 2.3 Intervention

#### 2.3.1 Physical activity

The physical activity component of the intervention consisted of home-based walking in conjunction with strength and conditioning exercises, which have been shown to be effective for CRF and also feasible and acceptable (Yeo et al., 2012). The overall aim of the intervention was to achieve a goal of 30 min of walking 3 times per week and 2–3 strength sessions, for major muscle groups per week by the end of the 8-week intervention (Donnelly et al., 2011). Weekly goals were suggested, and decided through collaboration with participants on an individual basis. Participants were able to set goals either in the mobile app or in a goal-setting diary. All participants received a weekly telephone call from the researcher (KMCC) using a standardised protocol and confidential information was stored on password protected devices. Telephone calls assessed any issues, set collaborative goals for the following week and captured any missing data that had been logged in the diary but not reported in the app.

#### 2.3.2 Mindfulness practice

The mindfulness intervention was based on the mindfulness-based stress reduction (MBSR) (Kabat-Zinn, 2013). It involved 8 weeks of home-based formal and informal mindfulness practice which included body scans, sitting, walking, and loving-kindness practice (Johns et al., 2015). Two mindfulness practices usually included in MBSR delivery are, the yoga and silent retreat, these were not included in the current interventions, as asynchronous delivery made these aspects difficult to facilitate, and few previous studies included them (Carlson et al., 2016).

#### 2.3.3 Digital procedure

**Fig. 1:**
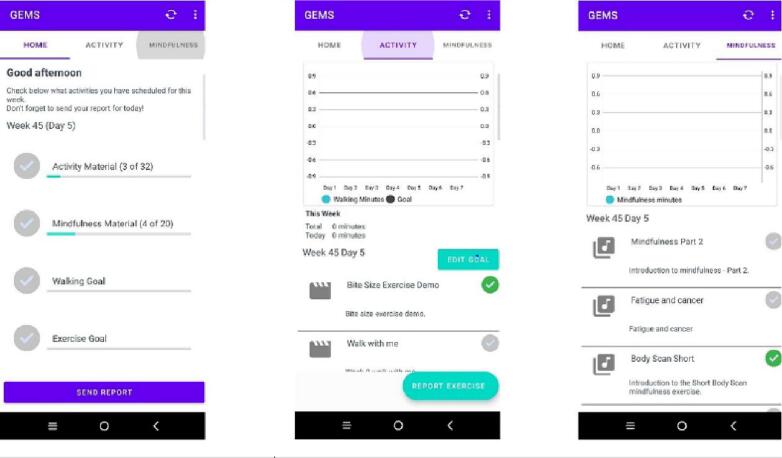
App screenshots.

Interventions were delivered via a bespoke mobile app developed and built by the research team. Participants received instructions on how to install the app and complete the registration process which permitted secure data collection (Cruciani et al., 2022). Once the app was downloaded, logged into and opened, participants could view a home page that gave them a running total of exercise/mindfulness materials accessed, and walking/exercise goals achieved. The home tab also had a ‘send report’ feature where participants uploaded reports of daily activity. The app was designed to take into account randomisation so participants were only able to view the content relevant to their allocated group. Data entered by participants was available to the study team via an administration portal which allowed for monitoring and feedback to participants (Fig. 1 app screenshots) (Cruciani et al., 2022).

#### 2.3.4 Outcome measures

Primary measures included the feasibility and acceptability of the study and were assessed in line with the MRC guidance on developing and evaluating complex interventions (Skivington et al., 2021). These included retention and adherence rates acceptability of interventions were assessed using focus groups which allowed participants to share their experiences on the virtual delivery, data collection and the exercise and mindfulness components of the intervention. Participant-reported outcome measures (PROMs) were collected for secondary outcomes through a battery of validated questionnaires.

The primary clinical outcome of CRF, was assessed using the Functional Assessment of Chronic Illness Therapy- Fatigue (FACIT-F), and a minimal clinically important difference of 3 points has been established for this measure (Sheehan et al., 2020). Lower scores with this measure indicated greater fatigue.

The secondary clinical outcomes were quality of life (QoL, Functional Assessment for Cancer Therapy-General (FACT-G)), anxiety and depression (Hospital Anxiety and Depression Scale (HADS)), sleep (The Pittsburgh Sleep Quality Index (PSQI)). Additionally, demographic information was collected at baseline on age, cancer type, stage, highest education level, income, and marital status.

### 2.4 Statistical analysis

As this was a feasibility study, means, standard deviation and median for attrition, adherence, and retention rates were calculated. Adherence was assessed by the total number of days completed divided by the total number of days the study ran for, expressed as a percentage. Attrition was reported as the number of participants dropping out for any reason, divided by the total number of participants randomised and expressed as a percentage. Retention was calculated by the number and proportion of participants completing the intervention, which was the number of weeks completed, divided by the number of possible weeks within the intervention (8 weeks) and expressed as a percentage. The retention rate for feasibility was set at 70 %, this was based on other similar studies that reported a retention rate of between 60 % and 100 %. (Gracey et al., 2021). Adherence rates were set at 67 % or two-thirds of exercise routine or protocol completed by participants (Hawley-Hague et al., 2016). Although this study was not powered to detect clinically significant changes in outcomes measures, data were collected on the outcomes of fatigue, depression, anxiety, sleep and HRQoL to determine whether intervention resulted in any change that would be feasible for use to inform a fully powered RCT.

### 2.5 Focus group analysis

Analysis of data from focus groups was completed using Thematic Analysis (Braun and Clarke, 2006). This type of analysis is flexible and allowed for the in-depth identification and interpretation of themes. (Castleberry and Nolen, 2018). The phases of the analysis included: familiarising with data, generating initial codes, searching for themes, reviewing themes, defining and naming themes and integrating these themes into a final report for publication (Braun and Clarke, 2006).

## 3 Results

**Fig. 2:**
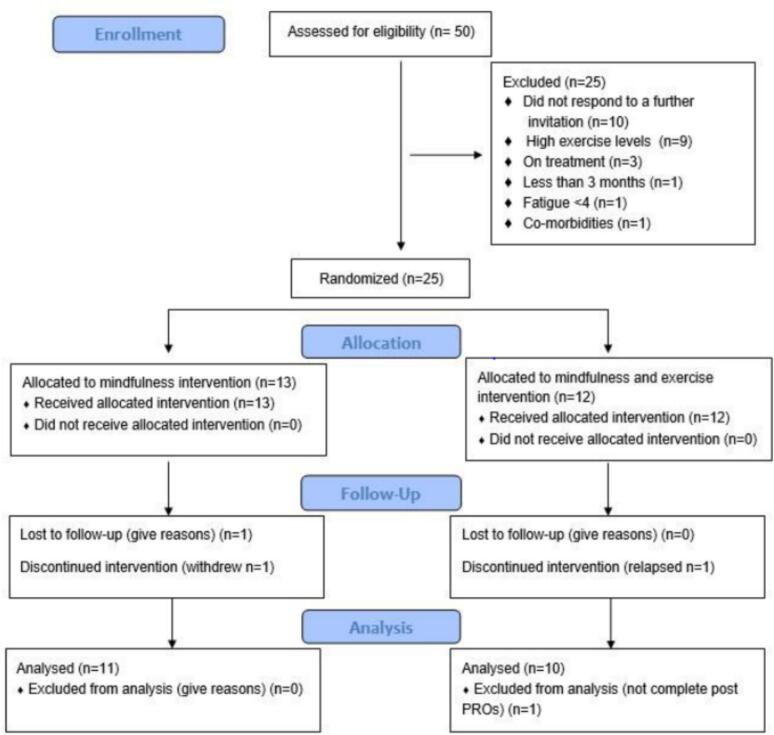
CONSORT diagram.

Out of 50 potential participants who expressed interest in the study, half did not meet the inclusion criteria, with the majority (n = 9, 36 %) screened as highly active, thus not meeting the sedentary definition in the inclusion criteria. The remaining reasons for non-inclusion were, currently on treatment (n = 3, 12 %), less than 3 months since completing treatment (n = 1, 4 %), not meeting the threshold for fatigue levels (n = 1, 4 %), and co-morbidities (n = 1, 4 %). The remaining 10 (40 %) did not respond to further study invitations (see CONSORT flow diagram Fig. 2). Twenty-five participants were recruited and successfully randomised over an 8-and-a-half-month period, giving an accrual rate of 2.9 participants per month.

### 3.1 Sample characteristics

The majority of the sample had a diagnosis of ovarian cancer (64 %) and were stage III (40 %) with similar numbers randomised to the two intervention groups. Most of the sample were white (N = 21, 84 %), the age range was 38 to 75 years, with a mean of 56.8 years (SD 9.18). Table 1 displays all the participant characteristics at baseline and by group assignment.

**Table 1:** participant characteristics.

Demo/Clinical Characteristics	Overall (n = 25)		Mindfulness and exercise (n = 12)		Mindfulness (n = 13)	
	No of participants	%	No of participants	%	No of participants	%
Age years						
Mean (SD)	56.8 (9.18)		54.5 (9.22)		59.1 (8.80)	
Range	38–75		38–67		42–75	
Ethnicity						
White-English/Welsh/Scottish/ Northern Irish/British	21	84	9	75	12	92
Black, African, Caribbean or Black British- African	1	4	0	0	1	8
Asian or Asian British-Indian	2	8	2	17	0	0
Other (Jewish)	1	4	1	8	0	0
Marital Status						
Married	13	52	5	42	8	61
Widowed	1	4	0	0	1	8
Divorced	4	16	2	17	2	15
Separated	1	4	0	0	1	8
Never married	5	20	4	33	1	8
No answer	1	4	1	8	0	0
Type of Gynae Cancer						
Ovarian	16	64	7	58	9	70
Endometrial/Uterine	3	12	3	25	0	0
Cervical	2	8	2	17	0	0
Vaginal	2	8	0	0	2	15
Ovarian & Endometrial	2	8	0	0	2	15
Stages						
I	4	16	1	8	3	24
II	6	24	6	50	0	0
III	10	40	5	42	5	38
IV	2	8	0	0	2	15
I & II	2	8	0	0	2	15
III & IV	1	4	0	0	1	8
Employment						
Full-time	8	32	3	25	5	37
Part-time	6	24	5	42	1	8
Unemployed	2	8	1	8	1	8
Retired	7	28	3	25	4	31
Disabled	1	4	0	0	1	8
Unemployed & Disabled	1	4	0	0	1	8
Salary						
0–25000	6	24	2	17	4	30
25001–50000	8	32	3	25	5	38
50001–75000	4	16	3	25	1	8
75001–100000	1	4	0	0	1	8
>100000	1	4	1	8	0	0
Prefer not to say	5	20	3	25	2	16
Education						
Secondary school	2	8	0	0	2	15.4
Sixth form or equivalent	6	24	1	8	5	38.4
Bachelor’s degree	11	44	9	76	2	15.4
Master’s degree	2	8	0	0	2	15.4
Doctoral Degree	1	4	1	8	0	0
Other	3	12	1	8	2	15.4

### 3.2 Attrition and retention

The attrition rate was low with only three participants not completing the study following randomisation. Baseline outcome measures were completed by these participants, but no further data was collected. Reasons for withdrawal were not wanting to continue with the study (n = 1), relapse of disease (n = 1) and lost to follow-up (n = 1). Two of these participants were randomised to the mindfulness-only group and the other to the mindfulness and exercise group. Therefore, retention was 88 % (22/25), which filled the proposed criteria for retention/attrition rate in this study which was 70 %.

### 3.3 Adherence

The overall adherence for the current study was 72.72 %.

### 3.4 PROM

#### 3.4.1 Fatigue and patient reported outcomes

Table 2 provides the mean difference between groups on all outcomes at week 8 post intervention. The primary clinical outcome, FACIT-F, showed that both groups had a decrease in fatigue post-intervention. With the mindfulness and exercise group showing a slightly larger decrease than the mindfulness alone group. The secondary clinical outcomes also showed a decrease in mean difference for QOL (decrease equals improvement), anxiety and depression showed a larger decrease in mean difference for the mindfulness and exercise group, compared to the mindfulness alone group. For sleep both groups displayed a similar reduction in sleep outcome. The changes in mean score for CRF, QOL, and sleep for both groups reached MCID but only the mindfulness and exercise group reached a MCID for anxiety and depression.

**Table 2:** Outcome variables results.

Outcome Variable	Mean (SD)		
	Baseline	8 weeks	Mean Difference
FACIT Mindfulness & Exercise Group Mindfulness Alone Group	21.31 (3.84)23.65 (9.51)	35.42 (5.18)31.45 (7.90)	14.11 7..80
FACT-G Mindfulness & Exercise Group Mindfulness Alone Group	60.13 (7.48)52.45 (12.95)	70.53 (9.79)58.85 (10.31)	10.40 6.40
HADS (Anxiety) Mindfulness & Exercise Group Mindfulness Alone Group	10.27 (3.87)9.10 (3.38)	5.40 (2.17)6.20 (2.61)	−4.87 −2.90
HADS (Depression) Mindfulness & Exercise Group Mindfulness Alone Group	7.36 (3.35)7.36 (2.90)	3.10 (1.59)5.27 (2.57)	−4.26 −2.09
PSQI Mindfulness & Exercise Group Mindfulness Alone Group	11.11 (2.26)12.00 (5.91)	8.40 (3.86)9.50 (5.00)	−2.71 −2.50

#### 3.4.2 Physical activity adherence

Physical activity adherence was determined by participants self-reported minutes of activity through the app. Adherence was based on engaging in ≥30 min of aerobic exercise weekly for ≥2/3 of the trial. The walking minutes reported via the app showed that, more than two-thirds of the 11 participants who completed the study walked ≥30 min per week, demonstrated an adherence of 91.66 %. In addition, within this group, 50 % of participants met the required 150 min suggested by WHO.

#### 3.4.3 Mindfulness practice adherence

Adherence to the mindfulness component of an intervention has been identified in previous studies by the number of weeks of engagement. Optimally 4 weeks has been accepted as adequate for adherence (Park et al., 2020). Therefore, within the current study, daily self-reports from participants of having completed mindfulness for 4 weeks (50 %) was accepted as adherence to the intervention being reached. The, overall adherence to mindfulness in this current study was 81.81 %. Within the exercise and mindfulness arm, only 4 fell below the 50 % threshold, therefore 63.63 % adhered to the study. In the mindfulness only arm, no participant fell below the 50 % level therefore mindfulness-only achieved 100 % adherence.

#### 3.4.4 Focus group findings

Ten participants took part in one of three online focus groups and participants could have received either of the allocated interventions (mindfulness-only or mindfulness and exercise). Given that all participants had used an active intervention, it was felt that they could share the experience of their participation regardless of which group they were allocated to. Three main themes were identified, these were, benefits of participation, barriers to participation and digital delivery of the intervention. There was some scepticism before participation as to whether the intervention, particularly mindfulness would have any benefit, but there was general agreement that their experience was positive. Participants recounted that the perceived benefits of both interventions outweighed any barriers, with emotional benefits being the most commonly discussed. For participants in the mindfulness and exercise group, other benefits included physical fitness, changes in body shape and increased self-efficacy. The weekly telephone support call from the researcher was highlighted as positive and reaffirmed the continual need of healthcare professional (HCP) support for many months and years post-treatment. Both groups felt that they gained much from participation and that the intervention gave them the opportunity and motivation to resume activities, particularly the physical activities, participated in prior to diagnosis. The main barriers identified by both groups were time, lack of motivation, life events, and illness or treatment effects such as fatigue. The online format for delivery of this intervention was received positively with few issues and minimal negative feedback, apart from a few app related technical issues. Suggestions for improvements included the inclusion of peer support and notifying HCPs of this type of intervention so could be disseminated to other people living with and beyond cancer.

## 4 Discussion

This study assessed the feasibility and acceptability of delivering online interventions for managing CRF in women with gynaecological cancer. In terms of feasibility both interventions showed high adherence and retention rates, low attrition, improved PROMs and were deemed as acceptable to participants. Overall adherence in the current study for both groups combined was 72.72 %, but adherence to exercise using the walking minutes reported via the app was 91.66 %. This is in higher than the proposed target of 67 % of the intervention, which is in keeping with another study that also used home-based walking for ovarian cancer patients where a 91.6 % adherence rate was reported participants who walked for >30 min per week (Hawley-Hague et al., 2016, Zhou et al., 2017). Daily reports of mindfulness in the current study revealed that adherence was 81.81 %. This was higher than in previous feasibility studies that employed mindfulness for breast cancer where adherence was reported as 66.7 % (Salvador et al., 2022).

The digital delivery of the interventions was a unique aspect of this study and was viewed by participants as something positive that they could take part in without travelling to classes or being exposed to circulating viruses such as COVID-19 and flu. Also, participant engagement with the app was important for establishing feasibility of delivering interventions via this platform. The convenience and app content particularly in relation to the level of information was positively received by participants. Such a finding is in keeping with other studies, in that the use of online interventions in home settings has been seen as a facilitator for completing activities (Compen et al., 2017). However, it is important that app content is clear and pitched for the specific users as complex interventions such as exercise and mindfulness if not clear can lose the user and can result in a reduction in engagement and adherence (Compen et al., 2017). Therefore, suggesting that complex intervention delivery should be accompanied with clear instructions and if possible in multiple formats. Additionally, as was included in the current study a PPI advisory group could help guide the content.

Based on the primary clinical outcome of CRF, assessed by the FACIT-F score, mean scores improved from baseline to post intervention, which seemed to be more marked for the mindfulness and exercise group. The minimal clinically important difference (MCID), is 3 points for the FACIT-F (Chan et al., 2018). Both groups met this threshold, with a larger MCID observed in the mindfulness and exercise group. These findings align with those of Sheehan *et al.* who found an equivalent change for CRF in the exercise arm of their study (Sheehan et al., 2020). Other studies that have used exercise as an intervention to manage CRF and included the FACT-F have also demonstrated a positive post-intervention effect, with a change in mean scores ranging from 2.4 to 4.5. (Donnelly et al., 2011).

The use of mindfulness as an active comparator within this study appears to be unique, as most studies have used waitlist control (WLC) or usual care (UC). Previously used active comparators have been education, psychological support, cognitive behavioural therapy, and attention control (Huang et al., 2019). Few have used mindfulness, although Hwang *et al.* (2016) (Hwang et al., 2016) did incorporate relaxation within a home-based exercise programme for ovarian cancer survivors. However, the two studies that used mindfulness for CRF were able to demonstrate similar results post intervention to the current study (Salvador et al., 2022). With regard to active comparators a review of 21 studies that explored the effects of mindfulness based interventions on CRF in women found that studies that had WLC or UC often showed a larger effect for the intervention arm (McCloy et al., 2022). However, the types of active comparators often used in previous studies may not have evidence that they were effective for a particular symptom like CRF (McCloy et al., 2022). The current study used an active comparator that the European Society of Medical Oncology guidelines had recommended for CRF management. Whilst the current study did demonstrate an improvement in mean scored from baseline on CRF for both interventions, the mindfulness and exercise arm of the study appeared to show a larger response compare to mindfulness only. This suggests that the combination of two interventions had a better response and should be considered for future testing in a fully powered RCT.

Similar findings were apparent both groups in the secondary clinical outcome measures with HRQoL showing a mean change of >5 points, which would be considered a MCID (Jayadevappa et al., 2017). In addition, anxiety and depression improved for both groups, with the exercise and mindfulness group showing a larger change in the post intervention mean scores, possibly indicating the need for both interventions to be used concurrently to achieve the maximum results for participants. These results may indicate a reciprocal effect of these two interventions, where exercise may increase mindfulness and mindfulness may increase the effects of exercise training (Goldstein et al., 2020).

Qualitative feedback from participants in the current study indicated that they gained physical and emotional benefits from the interventions. Physically, participants within the mindfulness and exercise group felt that they experienced weight loss, increased in muscle tone and physical fitness. This is similar to the physical changes in fitness and weight loss reported by patients with endometrial cancer who participated in a 12-week exercise study and in muscular strength in relation in those with ovarian cancer (Rossi et al., 2016, Mizrahi et al., 2015). These outcomes are in keeping with cancer patients’ expectations of what participating in exercise can achieve. Psychological benefits were reported by all participants in the current study, irrespective of which arm of the study they were randomised to. The use of interventions such as mindfulness has showed an improvement in anxiety and depression with a reduction in mood disturbance, stress and an increase in emotional and functional QOL (Carlson et al., 2016). Furthermore, the use of mindfulness has been shown to help people to develop strategies for managing areas like stress and also the ability to re-perceive, which is described as changing the perception of how something is viewed (Madonna, 2018). This re-perceiving was evident within the current study as participants were able to describe that CRF was ‘*no longer the enemy’* and not something that inhibited them from participating in activities such as exercise. This is similar to the findings of Hoffman *et al.* in a study of 92 participants following an 8-week mindfulness programme (Hoffman et al., 2012).

The strengths of this study lay within the design which ensured that some issues in previous studies were addressed (Park et al., 2020). These included the randomisation, pilot testing, involvement of PPI, the use of validated outcome measures, the active comparator as the control arm and the online focus groups format. Unfortunately, due to time constraints, follow-up was not part of the original design for this study, hence it is not possible to comment on whether benefits were maintained post-intervention. This is an area for further exploration as it would provide insights into the long-term efficacy of interventions for this population and also whether participants require further interventional support that may be delivered through community-based providers to help maintain the original positive intervention outcomes.

Another limitation of this study is that participants were recruited through social media. Although this approach is efficient and offers clear advantages, it can introduce selection bias by over-representing certain age, ethnic and socioeconomic groups and by excluding individuals with limited digital literacy or restricted internet access.

This was a feasibility study and the sample size was small with most participants having been diagnosed with ovarian cancer, this is despite endometrial cancer being diagnosed at similar rates in the UK. Additionally, ovarian cancer often presents later, carrying a poorer prognosis, which may result in more severe symptoms and longer term effects, hence those with ovarian cancer may be more motivated to take part and actively seek out research to manage symptoms. Given these factors, a fully powered RCT is warranted to examine the effectiveness of these interventions on the symptoms of CRF, depression, anxiety, sleep and HRQoL.

## 5 Conclusion

In conclusion this study demonstrated the feasibility of delivering online interventions for the management of CRF in a gynaecological cancer sample. Not only was acceptability and satisfaction reported by participants, but outcome measures also indicated positive trends in reaching MCID for symptoms, following interventions. The use of apps in healthcare is not only more common, but also more expectant of patients and healthcare professionals. Ensuring that engagement and continual renewal of content and delivery is maintained this platform of delivery maybe the future of healthcare.

## Role of the funding source

This study was undertaken as part of a PhD studentship (KMCC) at Ulster University and funded by the Department of the Economy (DfE) studentship. The funders did not have a role in study design, data collection and analysis, decision to publish or preparation of the manuscript.

## CRediT authorship contribution statement

**Kairen McCloy:** Writing – review & editing, Writing – original draft, Resources, Project administration, Methodology, Investigation, Conceptualization. **Ciara Hughes:** Writing – review & editing, Writing – original draft, Supervision, Resources, Project administration, Methodology, Funding acquisition, Conceptualization. **Lynn Dunwoody:** Writing – review & editing, Writing – original draft, Supervision, Resources, Project administration, Methodology, Funding acquisition, Conceptualization. **Joanne Marley:** Writing – review & editing, Writing – original draft, Supervision, Resources, Project administration, Methodology, Funding acquisition, Conceptualization. **Ian Cleland:** Writing – review & editing, Software, Methodology, Conceptualization. **Federico Cruciani:** Writing – review & editing, Software, Methodology, Conceptualization. **Jackie Gracey:** Writing – review & editing, Writing – original draft, Supervision, Resources, Project administration, Methodology, Funding acquisition, Conceptualization.

## Declaration of competing interest

The authors declare that they have no known competing financial interests or personal relationships that could have appeared to influence the work reported in this paper.
